# Identification of antiplasmodial triterpenes from *Keetia* species using NMR-based metabolic profiling

**DOI:** 10.1007/s11306-019-1487-4

**Published:** 2019-02-21

**Authors:** Rafael Teixeira Freire, Joanne Bero, Claire Beaufay, Denise Medeiros Selegato, Aline Coqueiro, Young Hae Choi, Joëlle Quetin-Leclercq

**Affiliations:** 1 Natural Products Laboratory, Institute of Biology, Leiden University, Sylviusweg 72, 2333 BE Leiden, The Netherlands; 2 Pharmacognosy Research Group, Louvain Drug Research Institute, Université catholique de Louvain, UCLouvain, Avenue E. Mounier, 72, B1.72.03, B- 1200 Brussels, Belgium; 3 College of Pharmacy, Kyung Hee University, Seoul, 02447 Republic of Korea

**Keywords:** *Keetia leucantha*, *Keetia venosa*, In vitro antiplasmodial activity, NMR-based metabolomics, Triterpenes, STOCSY

## Abstract

**Introduction:**

The increase in multidrug resistance and lack of efficacy in malaria therapy has propelled the urgent discovery of new antiplasmodial drugs, reviving the screening of secondary metabolites from traditional medicine. In plant metabolomics, NMR-based strategies are considered a golden method providing both a holistic view of the chemical profiles and a correlation between the metabolome and bioactivity, becoming a corner stone of drug development from natural products.

**Objective:**

Create a multivariate model to identify antiplasmodial metabolites from ^1^H NMR data of two African medicinal plants, *Keetia leucantha* and *K. venosa*.

**Methods:**

The extracts of twigs and leaves of *Keetia* species were measured by ^1^H NMR and the spectra were submitted to orthogonal partial least squares (OPLS) for antiplasmodial correlation.

**Results:**

Unsupervised ^1^H NMR analysis showed that the effect of tissues was higher than species and that triterpenoids signals were more associated to *Keetia* twigs than leaves. OPLS–DA based on *Keetia* species correlated triterpene signals to *K. leucantha*, exhibiting a higher concentration of triterpenoids and phenylpropanoid-conjugated triterpenes than *K. venosa*. In vitro antiplasmodial correlation by OPLS, validated for all *Keetia* samples, revealed that phenylpropanoid-conjugated triterpenes were highly correlated to the bioactivity, while the acyclic squalene was found as the major metabolite in low bioactivity samples.

**Conclusion:**

NMR-based metabolomics combined with supervised multivariate data analysis is a powerful strategy for the identification of bioactive metabolites in plant extracts. Moreover, combination of statistical total correlation spectroscopy with 2D NMR allowed a detailed analysis of different triterpenes, overcoming the challenge posed by their structure similarity and coalescence in the aliphatic region.

**Electronic supplementary material:**

The online version of this article (10.1007/s11306-019-1487-4) contains supplementary material, which is available to authorized users.

## Introduction

Every year several 100 million of people get malaria, 1.2 million of which die. Even if a reduction of 37% cases has been recently reported, it remains the most severe parasitic disease worldwide, particularly for children under the age of five and pregnant women (World Health Organisation (WHO) 2018). Despite efforts to develop vaccines, the antigenic variability of these parasites allows only a partial and decreasing protection against clinical malaria and only in children and infants (Olotu et al. 2016).

In addition, malaria therapy faces some emerging problems such as parasite multidrug resistance, mosquito resistance to insecticides and the shortage of the time and money required for the development of new synthetic and natural leads (Achan et al. 2018; Woodrow and White 2017). The need to discover new prototypes of drugs is thus very important. In this context, natural products provide a high degree of lead-/drug-similarity, remaining undoubtedly the best source of native drugs or structural templates for antimalarial compounds development (Cargnin et al. 2018; Da Silva et al. 2013). A recent review reporting the new drugs available on the market during the last 34 years showed that approximately 60% of these new antiparasitic drugs have a natural origin/pharmacophore (Newman and Cragg 2016).

To date, the natural product database NAPRALERT has reported over 152 plant genera with a historical record of antimalarial properties, thus offering unlimited possibilities for the identification of novel hits/targets (Graham and Farnsworth 2010; Mojab 2012; Wells 2011). So far, however, the chemical and biological potential of these medicinal plants remains little explored.

When attempting to screen plant-matrixes, the large dynamic range and diversity of metabolites still hamper their identification and biological correlation. In the past, bio-guided fractionation was often used to identify active compounds. However, these reductionist procedures often lead to a laborious, time-consuming, non-comprehensive and expensive process of isolation and purification (Chen et al. 2015; Pezzuto 1997). Recently, metabolomics emerged as a fast alternative screening method to correlate chemical and biological data of natural products (Yuliana et al. 2011). Moreover, in an untargeted analysis of medicinal plants, metabolomics allows a broad range of chemical comparison between samples (Queiroz et al. 2009), prioritizing the identification of active molecules and, consequently, improving the process of chemotype and sample selection (Abreu et al. 2017; Kumar et al. 2014; Stermitz et al. 2000).

Considering their coverage of metabolites, sensitivity and resolution, NMR and MS methodologies are currently the most popular methods and are complementarily used for detailed chemical information on many biological systems. Mass spectrometry offers high sensitivity and resolution with some structure information deduced from accurate mass, fragmentation patterns or isotope distribution (Lei et al. 2011; Lindon et al. 2007; Markley et al. 2017). On the other hand, NMR provides a highly reproducible and non-destructive analysis with minimal sample preparation, enabling the detailed elucidation of a wide range of metabolic groups, including isomers and compounds that are difficult to ionize or derivatize for MS (Markley et al. 2017).

NMR-based chemical profiling has often been used in plant-metabolomics for chemotaxonomy (Kim et al. 2010a), quality control (Yang et al. 2006) and as a bioactivity screening method (Cardoso-Taketa et al. 2008; Yuliana et al. 2011) following a suggested protocol (Kim et al. 2010a). In addition to simple one-dimensional ^1^H NMR analysis, various two-dimensional experiments can be applied to ensure a broad interpretation of the data, providing detailed structure elucidation and quantification (Brennan 2014; Markley et al. 2017; Nagana Gowda and Raftery 2015). However, the difficulty of hyphenating NMR to chromatographic separation systems and its low-sensitivity has led to some inherent problems in chemical profiling research, including spectral congestion and the identification of minor metabolites (Kim et al. 2010b). For example, the elucidation of selected ^1^H resonances in the congested region, requires a previous signal deconvolution (Emwas 2015). Two-dimensional NMR spectra, e.g. J-resolved (Huang et al. 2015; Ludwig and Viant 2010) and ^1^H–^13^C-HSQC (Öman et al. 2014; Xi et al. 2008) have been applied to the analysis of complex spectra, in which an additional axis provides a higher resolution that can reveal invisible signals or peak purity. Furthermore, recent advances for overcoming these highly coalescence signals can also be based on the use of post-analytical deconvolution algorithms, assisting on detection and quantification of metabolites without the use of separation techniques. One example is statistical total correlation spectroscopy (STOCSY), which enables digital separation not limited to the usual connectivity of two-dimensional NMR methods and takes advantage of the multicollinearity of the signals intensity in a ^1^H NMR spectra (Cloarec et al. 2005).

In this study, NMR-based metabolomics with STOCSY was applied to a model of an antiplasmodial medicinal plant in order to identify bioactive metabolites involved in the activity. Recently, dichloromethane extracts of *Keetia leucantha*, an African antimalarial plant listed by the *Direction de la Protection Sanitaire* of the Benin Ministry of Health, showed promising in vitro antiplasmodial activity against both susceptible and resistant *Plasmodium* strains and in vivo antimalarial efficacy without any acute toxicity at therapeutic doses. This activity has been related to active triterpenes (Beaufay et al. 2017, 2019; Bero et al. 2009, 2013). In this paper, we report the use of NMR-based metabolomics and the implementation of a post-analytical deconvolution algorithm to identify antiplasmodial metabolites from ^1^H NMR data of *K. leucantha* and another related species, *K. venosa*. Specifically, dichloromethane extracts of twigs and leaves of both *Keetia* species were measured by ^1^H NMR and the spectra were submitted to orthogonal partial least square modeling (OPLS). Correlation with bioactivity was performed according to their in vitro antiplasmodial response (expressed by IC_50_) against chloroquine sensitive and -resistant *Plasmodium falciparum* strains (3D7 and W2, respectively). For metabolite elucidation, targeted OPLS signals were submitted to STOCSY and confirmed by 2D-NMR experiments.

## Materials and methods

### Plant material

*Keetia venosa* (Oliv.) Bridson (17 twigs and 17 leaves) and *K. leucantha* (K. Krause) Bridson (27 twigs and 27 leaves) (Rubiaceae) were collected by Dr Agbani, a specialized botanist from University of Abomey-Calavi in Benin over the period of 2014–2015 in the Republic of Benin, West Africa, in different geographical locations in Donga or Ouémé/Zou departments, respectively: Djougou, Bassila and Belefunga for *K. venosa* and Adjara, Tako (Porto Novo), Lama and Katagon for *K. leucantha*.

The leaves and twigs samples were air-dried immediately after collection. Herbarium samples, prepared locally during the collection, and further identified by Dr. Olivier Lachenaud, compared with voucher specimens, have been deposited at the Herbarium of the National Botanic Garden of Belgium (Vouchers BR0000005087129 and BR00000014420382 for *K. leucantha* and BR0000005087228, BR0000005087242, BR00000014420443 and BR00000014420375 for *K. venosa*).

### Sample preparation

Dried and ground plant material (200 mg) was vortexed and ultrasonicated for 30 s and 15 min, respectively, with 2.0 mL of dichloromethane. For NMR- and biological analysis, the filtered (with cotton wool) and dried dichloromethane extracts were dissolved in 750 µL of CD_3_OD, vortexed for 30 s, ultrasonicated for 1 min and centrifuged for 20 min at 13,000 rpm. The supernatants were divided in two solutions (300 µL) for NMR and in vitro antiplasmodial assay.

### NMR experiments

^1^H NMR spectra of the plant samples were acquired on a Bruker 600 MHz Advance II spectrometer (Bruker, Germany) equipped with a 5 mm triple resonance inverse cryoprobe and a z-gradient system. Prior to data acquisition, automatic tuning and matching of the probe was performed, as well as manual shimming and automatic proton pulse calibration (*pulsecal*, Bruker).

^1^H NMR analysis was performed in 3 mm tubes and acquired by water 1D-water presaturation pulse sequence with composite pulses (zg30pr, Bruker) at the following parameters time domain (TD) 32 k; number of scans (NS) 64; spectral width (SW) 20 ppm; water signal irradiation point (o1) 4.84 ppm; temperature 298 K and relaxation delay (d1) 1.5 s.

For two dimensional-NMR acquisition, (1) gradient-selected heteronuclear single quantum coherence (HSQC) was performed by phase-sensitive ge-2D multiplicity edited HSQC using PEP and adiabatic pulses with gradients in back-inept, (2) gradient-selected heteronuclear multiple bond correlation (HMBC) was acquired by phase-sensitive ge-2D HMBC using a two-fold low-pass J-filter; and (3) J-resolved measurement was performed using a standard pulse sequence with 25 Hz CW-based water signal suppression. For HMBC and HSQC acquisition, each parameter of ^1^H (f2) and ^13^C (f1) were as follows: frequency 600.13 and 150.92 MHz, time domain (TD) 2 k and 512 increments, spectral width (SW) 10 and 230 ppm, number of scans (NS) 64, relaxation delay (d1) 1.00 s and measuring temperature 298 K. For J-resolved acquisition, operating frequency, TD and SW were 600.13 MHz, 16 k, and 20 ppm for both axis (f1 and f2), respectively. Number of scans (NS) was 8; relaxation delay (d1) 2.00 s and temperature 298 K. The long-range coupling constant used for HMBC was 8.0 Hz.

### In vitro antiplasmodial activity

In vitro antiplasmodial activity was evaluated based on parasite viability using the lactate dehydrogenase assay (Makler et al. 1993) on chloroquine-sensitive 3D7 and chloroquine-resistant W2 *P. falciparum* strains (Murebwayire et al. 2008). Artemisinin (Sigma-Aldrich, Overijse, Belgium) was used as a positive control in all experiments, with an initial concentration of 100 ng/mL. Tests were performed as described by Bero et al. (2013) with a minor modification: extracts were tested in eight-serial two-fold dilutions (concentration range: 0.78–100 µg/mL, two wells/concentration). A statistical analysis was performed on GraphPad Prism 7.00 to compare activities of all *Keetia* extracts. As distributions were not Gaussian (according to D’Agostino-Pearson and Shapiro–Wilk normality tests), twigs and leaves extracts from *K. leucantha* and *K. venosa* were all compared to each other with a non-parametric ANOVA (Kruskal–Wallis and Dunn’s post-test, significance level of 0.05).

### Data processing and multivariate analysis

The ^1^H NMR spectra were automatically reduced to ASCII files. Spectral intensities were scaled to total intensity and reduced to integrated regions of equal width (0.04 ppm) corresponding to the region of δ 0.0–10.0 by AMIX software (Bruker). The regions of δ 4.7–4.9 and δ 3.28–3.34 were excluded from the analysis because of the residual signal of D_2_O and CD_3_OD, respectively.

The final processed data was exported in comma-separated values (.csv) and imported to SIMCA (version 15.2) software (Umetrics, Umeå, Sweden) for multivariate data analysis. All chemometric analysis were performed on the processed ^1^H NMR data using unit variance (UV) scaling method. The OPLS–DA analysis was modeled based on (1) *Keetia* species (*K. leucantha* and *K. venosa*) and OPLS analysis was modeled on (2) in vitro antiplasmodial activity (IC_50_ values) of all *Keetia* samples. For metabolite elucidation, STOCSY was applied to all targeted bioactive buckets selected from the OPLS loadings (Cloarec et al. 2005). Chemical connectivity of the targeted bucket (driver peak) was analyzed by STOCSY correlation and covariance algorithm using MATLAB R2017a software (Mathworks, Natick, MA, USA). For confirmation and individual compound assessment, 2D NMR experiments (J-resolved, HMBC and HSQC) were acquired and interpreted using MestreNova 12.0.3 software17 (MestreLab Research SL, Santiago de Compostela, Spain).

## Results and discussion

### Metabolic profiling of leaves and twigs of *Keetia* species by ^1^H NMR and multivariate data analysis

**Fig. 1:**
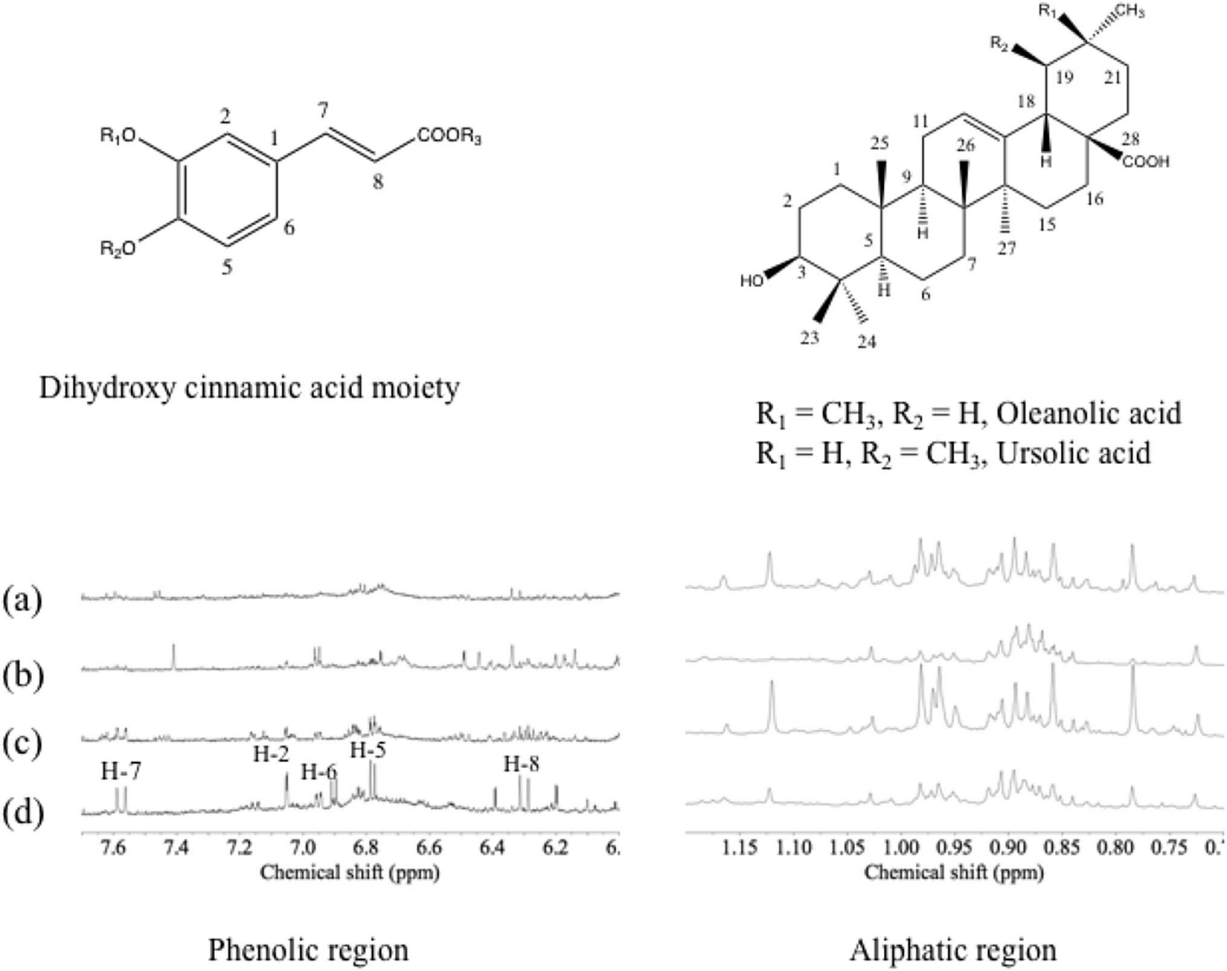
Chemical structures of dihydroxy cinnamic acid and triterpenoidal (oleanolic- and ursolic acids) moieties identified in *Keetia* species, and typical ^1^H NMR spectra (600 MHz, CH_3_OH-*d*_*4*_) of CH_2_Cl_2_ extracts of *K. venosa* twigs (a) and leaves (b), and *K. leucantha* twigs (c) and leaves (d) in phenolic (δ 6.0–7.7) and aliphatic region (δ 0.7–1.2). H-2, H-5, H-6, H-7 and H-8 are H of dihydroxy cinnamic acid moiety

^1^H NMR metabolic fingerprinting was applied as a high-throughput method for detecting chemical differences between *Keetia* samples and their related tissues. Figure 1 illustrates some representative ^1^H NMR spectra of CH_2_Cl_2_ extracts of *K. leucantha* and *K. venosa* (both leaves and twigs). Two major metabolic groups were detected in the spectra, triterpenoids and phenylpropanoids. In the region of δ 0.7–1.2, many characteristic CH_3_ resonances were found including H-23, H-24, H-25, H-26, H-27, H-29 and H-30 from the triterpenoids moiety. Moreover, in the region of δ 6.1–7.7, characteristic signals of dihydroxy cinnamic acid analogues, such as caffeic- or ferulic acids, were clearly detected in some *Keetia* samples.

Previous reports indicate that phenylpropanoids are present in both free form or conjugated to triterpenoids (Bero et al. 2013) and ^1^H NMR data suggest that both free- and phenylpropanoid-conjugated triterpenes are present in these species. Phenylpropanoid conjugated triterpenes were found to be more abundant in *K. leucantha* twigs than in any other samples (Bero et al. 2013).

**Fig. 2:**
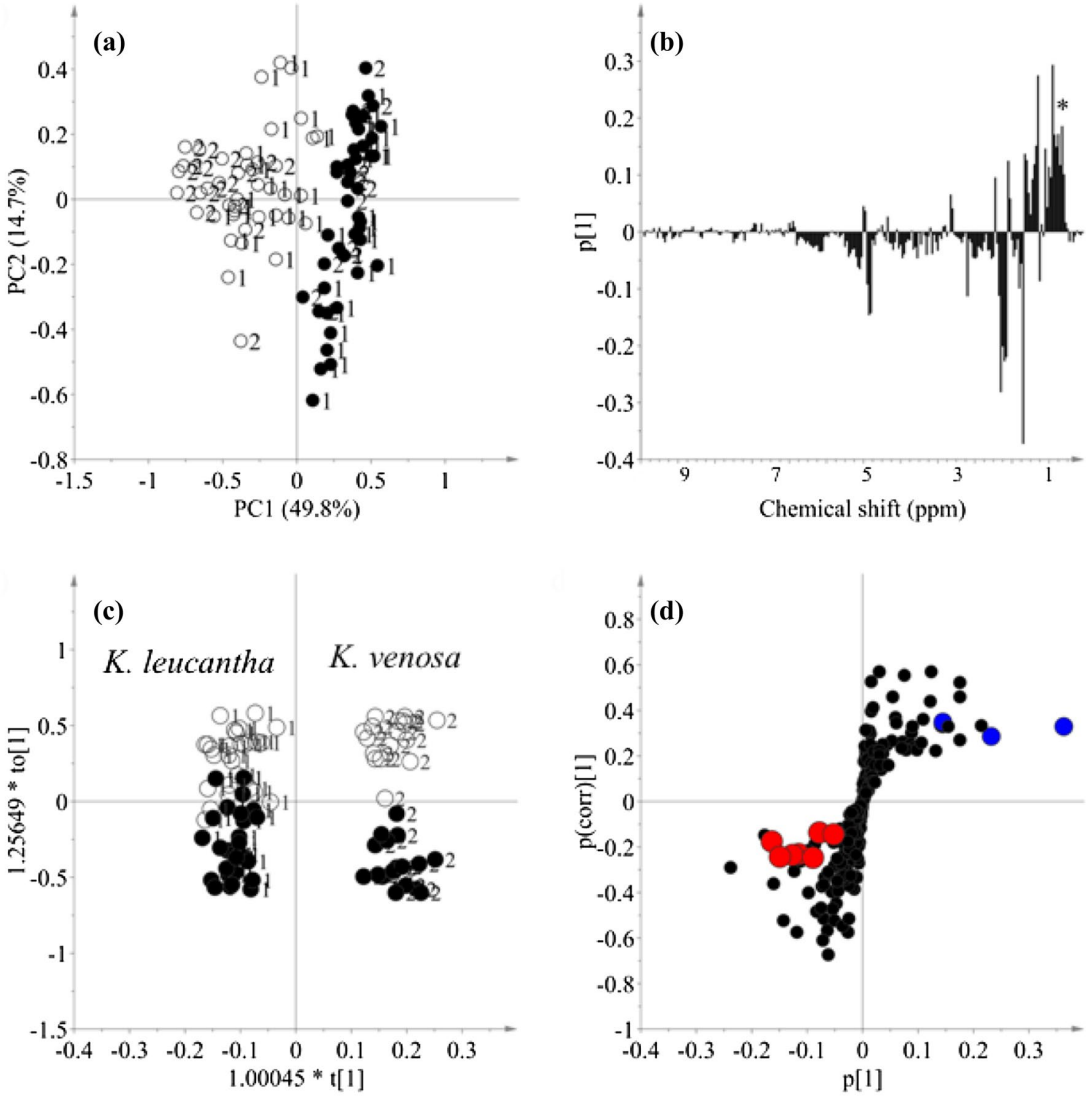
Score plot (PC1 × PC2) (**a**) and PC1 loading plot (**b**) of principal component analysis of *K. leucantha* and *K. venosa* samples (leaves and twigs), and OPLDS–DA score plot (**c**) (t1/to1) and S-plot (**d**) using two species classes. 1: *K. leucantha*, 2: *K. venosa*, o: Leaves, •: twigs. *: methyl signals of triterpenoids. Red (•) and blue dots (•) in (d) are methyl signals of triterpenoids and squalene, respectively

To overview the metabolites of leaves and twigs of the two *Keetia* species, ^1^H NMR spectra of 88 samples were further analyzed by PCA, as shown in Fig. 2. This raw ^1^H NMR data (bucketed) as well as their activity against two cell lines are in the Supplementary Table 1. The score plot of PCA in Fig. 2a showed that the effect of tissues on the metabolome was higher than that of species effect. The loading plot of the PCA was analyzed to identify the metabolites responsible for this discrimination between tissues. The characteristic methyl signals of triterpenoids were correlated with the positive side of PC1 loading plot in Fig. 2b, which indicates that the twigs from *Keetia* species were found to have higher level of triterpenoids than leaf samples.

Although the metabolic influence of species was much lower than that of tissues in PCA and not detected well using PC1 and PC2, the separation between the species might be analyzed in minor PCs. To investigate in detail the effect of species, a supervised multivariate data analysis, OPLS–DA was applied to the same data set using two classes of species (1: *K. leucantha* and 2: *K. venosa*). The score plot of OPLS–DA (Fig. 2c) showed a very clear separation and the model was well validated by permutation and CV–ANOVA tests (Q2 value > 0.85 in permutation test and p value in CV–ANOVA = 1.4126e^−29^). The S-plot (Fig. 2d) revealed that the methyl ^1^H resonances of triterpenoids were higher in *K. leucantha* samples than *K. venosa*. In addition, some aromatic resonances at δ 7.56 (*d, J* = 15.9 Hz), δ 7.05 (*d, J* = 2.1 Hz) and δ 6.29 (*d, J* = 15.9 Hz) were found to be more correlated with *K. leucantha* and were elucidated as the dihydroxy cinnamic acid moieties of triterpenic esters, previously reported in this species (Bero et al. 2013). On the other hand, signals at δ 1.59 (*s*), δ 1.60 (*s*), δ 2.07–2.09 (*m*), δ 5.09 (*t, J* = 7.2 Hz) and δ 5.11 (*t, J* = 7.2 Hz) were attributed to *K. venosa* samples and elucidated as an acyclic triterpene, squalene, a common secondary metabolite from the leaves of *Keetia* species (Bero et al. 2013; Thimmappa et al. 2014).

### In vitro antiplasmodial activity of *Keetia* samples and correlation with metabolomics data by OPLS

To assess the antiplasmodial potential of the twigs and leaves, all *Keetia* samples were tested in vitro for their antiplasmodial activity on chloroquine-sensitive (3D7) and chloroquine-resistant (W2) strains of *P. falciparum*. The mean IC_50_ of leaves and twigs extracts from *K. leucantha* were 124.5 ± 152.4 µg/mL and 77.9 ± 40.6 µg/mL on 3D7 strain, respectively, and 63.8 ± 40.6 µg/mL and 88.7 ± 50.7 µg/mL on W2 strain, respectively. In the case of *K. venosa*, the mean IC_50_ for leaves and twigs extracts were 129.2 ± 144.1 µg/mL and 207.4 ± 167.6 µg/mL on 3D7 strain, respectively, and 90.7 ± 98.5 µg/mL and 129.9 ± 92.7 µg/mL on W2 strain, respectively. Overall, *K. leucantha* showed similar to higher antiplasmodial activity on both strains than *K. venosa* with significant differences between both species twigs, *K. leucantha* leaves and *K. venosa* twigs on 3D7 (p < 0.01) but only between leaves of *K. leucantha* and twigs of *K. venosa* on W2 (p < 0.05). However, these activities are quite lower than artemisinin ones (IC_50_ = 0.008 ± 0.002 and 0.004 ± 0.001 µg/mL on 3D7 and W2 strains respectively) or previously reported activities for chloroquine (IC_50_ = 0.02 ± 0.01 and 0.49 ± 0.15 µg/mL on 3D7 and W2 strains respectively) (Bero et al. 2009). Nevertheless, crude extracts are mixtures of hundreds of compounds, some of which may have high activity, diluted with other non-active compounds. In the case of tissues among the same species, there was not much difference and the activity depended on the tested sample. Moreover, there was a high variation in the measured IC_50_, even in the same species and tissue. In particular, *K. venosa* samples showed a broader range of antimalarial results according to the harvest region and tissues (IC_50_ from 29.18 µg/mL to estimated 734.86 µg/mL for *P. falciparum* 3D7 and from 30.38 µg/mL to estimated 439.29 µg/mL for *P. falciparum* W2).

**Fig. 3:**
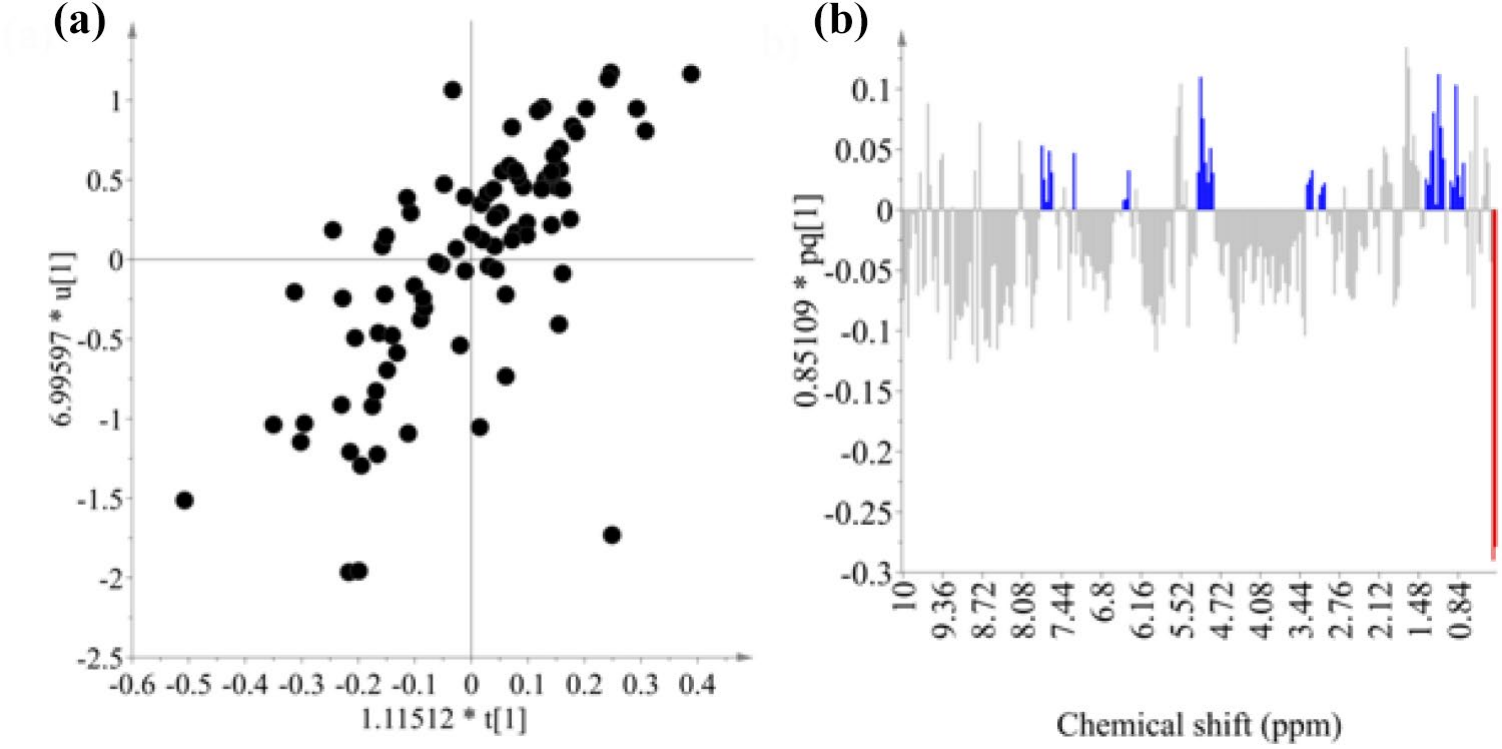
Score (**a**) and loading plot (**b**) of OPLS modelling with log IC_50_ of in vitro antiplasmodial assay against 3D7 and W2 *P. falcifarum* strains t1 of ^1^H NMR data versus u1 of log IC_50_ of in vitro antiplasmodial activity. r^2^ for the correlation = 0.487. Red bars in (**b**) are IC_50_ values against 3D7 and W2 *P. falcifarum* strains and blue bars in (**b**) are ^1^H resonances of triterpenoids and phenylpropanoids associated with the activity

As a next step, ^1^H NMR data of the samples were correlated with IC_50_ against both strains by OPLS modeling with two Y variables (IC_50_ on 3D7 and W2 strains). To optimize the model, two scaling methods (UV and Pareto) with Log transformation were evaluated. As shown in Table 1, the highest Q2 value was obtained from permutation test (100 permutations) when UV scaling with log transformation of Y variables were used. A strong correlation between metabolomics and antiplasmodial data was found in the score plot of OPLS modeling as shown in Fig. 3a, in which ^1^H NMR data and antiplasmodial activity against both *P. falciparum* strains were used as X- and Y-variables with UV scaling method.

**Table 1:** Q2 value of OPLS modeling of ^1^H NMR metabolomics data and antiplasmodial activity (IC_50_) against 3D7 and W2 *P. falciparum* strains using different scaling methods and transformation obtained from permutation test with 100 permutations

Scaling method	Transformation of IC_50_ (Y-data set)	Q2 value	
		3D7 strain	W2 strain
Unit variance	No	0.277	0.275
	Log	0.479	0.473
Pareto	No	0.145	0.099
	Log	0.314	0.323

To identify metabolites responsible for the activity, a loading plot was used as shown in Fig. 3b. Some characteristic signals of triterpenoids such as methyls, H-5 around δ 3.2 and H-12 around δ 5.2 were clearly related with the activity (negatively correlated with IC_50_ values). In addition to triterpenoidal signals, some characteristic ^1^H resonances of phenylpropanoids were found to be associated to activity (Fig. 3b). In a previous report on the metabolites presented in *K. leucantha*, free forms of triterpenoids and their phenylpropanoid-conjugates were found to be major secondary metabolites, but only phenylpropanoid-conjugates displayed high antiplasmodial activity and low cytotoxicity on WI38 cells (Bero et al. 2013). Moreover, loading plot of samples with low antiplasmodial activity were also correlated to triterpenoids signals, such as methyls at around δ 1.60, characteristic of the acyclic triterpene squalene (Fig. 3b).

Pentacyclic triterpenes have been already reported to show promising antimalarial potential (Beaufay et al. 2018; Isah et al. 2016) and some of them display high antiplasmodial activity and low cytotoxicity alone, in mixtures or in combination with artemisinin (Cargnin et al. 2018; Da Silva et al. 2013; Ma et al. 2008; Phillipson and O’Neill 1987). They also possess various pharmacological effects and are thought to act as multi-target compounds (Moneriz et al. 2011). Phenylpropanoid-conjugated triterpenes from *K. leucantha* have been shown to have higher antiplasmodial activity than their corresponding acids (Bero et al. 2013).

### Structure elucidation of ferulic acid conjugated triterpenoid esters

To elucidate the detailed chemical structure of the metabolites, several 2D-NMR such as J-resolved, COSY, HSQC and HMBC (Supplementary Figs. 1, 2, 3 and 4) were firstly applied to the signals selected by OPLS modeling. The identity of each moiety of triterpene (e.g. oleanolic acid and ursolic acid) and phenylpropanoid (e.g. ferulic acid) was confirmed by 2D-NMR experiments and comparison with the spectra of reference compounds. However, 2D-NMR spectrum was insufficient to determine the conjugation, if any, between the two moieties, that requires the detection of a correlation band e.g. H-27 and C=O in HMBC, in a very crowded region. To overcome this connectivity issue, STOCSY was used to connect the spin systems by the multicolinearity of the intensity of their signals along a set of spectra. By selecting a driver peak, also known as target peak, the STOCSY calculates the covariance and the correlation between all peaks in a dataset and creates a pseudo NMR spectrum, in which the intensities are related to the covariance values and the colors are related to the correlation values. Peaks from the same molecule or the same pathway to the driver peak are expected to have similar values, thus allowing their identification even in highly complex mixtures.

**Fig. 4:**
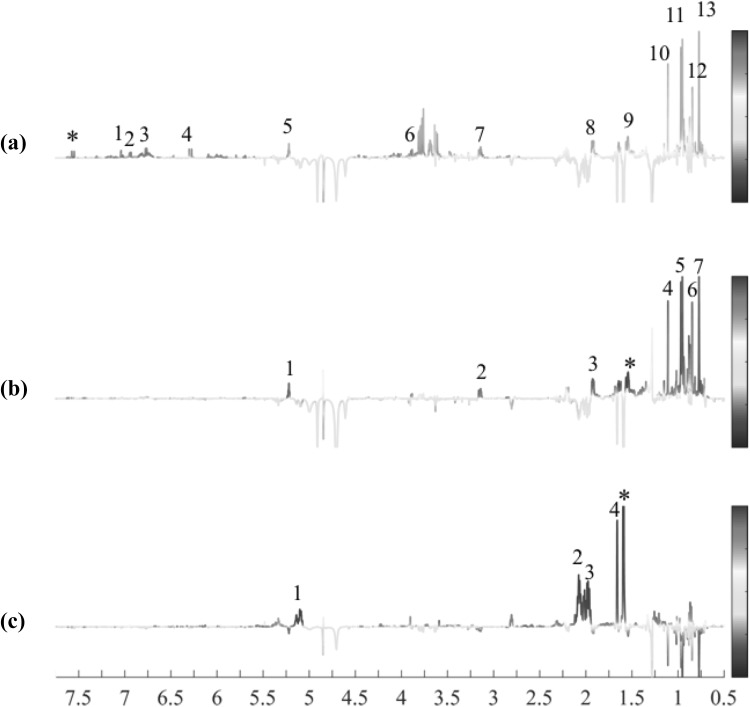
STOCSY plot using drivers peak at δ 7.55 (**a**), δ 1.56 (**b**) and δ 1.59 (**c**). Signal assignments; (**a**) *driver peak at δ 7.55 (H-7 of ferulic acid moiety), 1: H-2 of ferulic acid moiety, 2: H-6 of ferulic acid moiety, 3: H-5 of ferulic acid moiety, 4: H-8 of ferulic acid moiety, 5: H-12 of ursolic- and oleanolic acid, 6: OCH_3_ of ferulic acid moiety, 7: H-3 of ursolic- and oleanolic acid, 8: H-11 of ursolic- and oleanolic acid, 9: H-6 of ursolic- and oleanolic acid, 10: H-27 of ursolic- and oleanolic acid, 11: H-25 of ursolic- and oleanolic acid, 12: H-26 of ursolic- and oleanolic acid, 13: H-24 of ursolic- and oleanolic acid. (**b**) *Driver peak at δ 1.56 (H-6 of ursolic- and oleanolic acid), 1: H-12 of ursolic- and oleanolic acid, 2: H-3 of ursolic- and oleanolic acid, 3: H-11 of ursolic- and oleanolic acid, 4: H-27 of ursolic- and oleanolic acid, 5: H-25 of ursolic- and oleanolic acid, 6: H-26 of ursolic- and oleanolic acid, 7: H-24 of ursolic- and oleanolic acid. (**c**) *Driver peak at δ 1.59 (CH_3_ attached to C-6 of squalene), 1: H-3, H-7 and H-11 of squalene, 2: H-4 and H-8 of squalene, 3: H-1 and H-9 of squalene, 4: H-1 and H-2 of squalene. For the ^1^H assignments see the chemical structures in Figs. 1 and 5

**Fig. 5:**
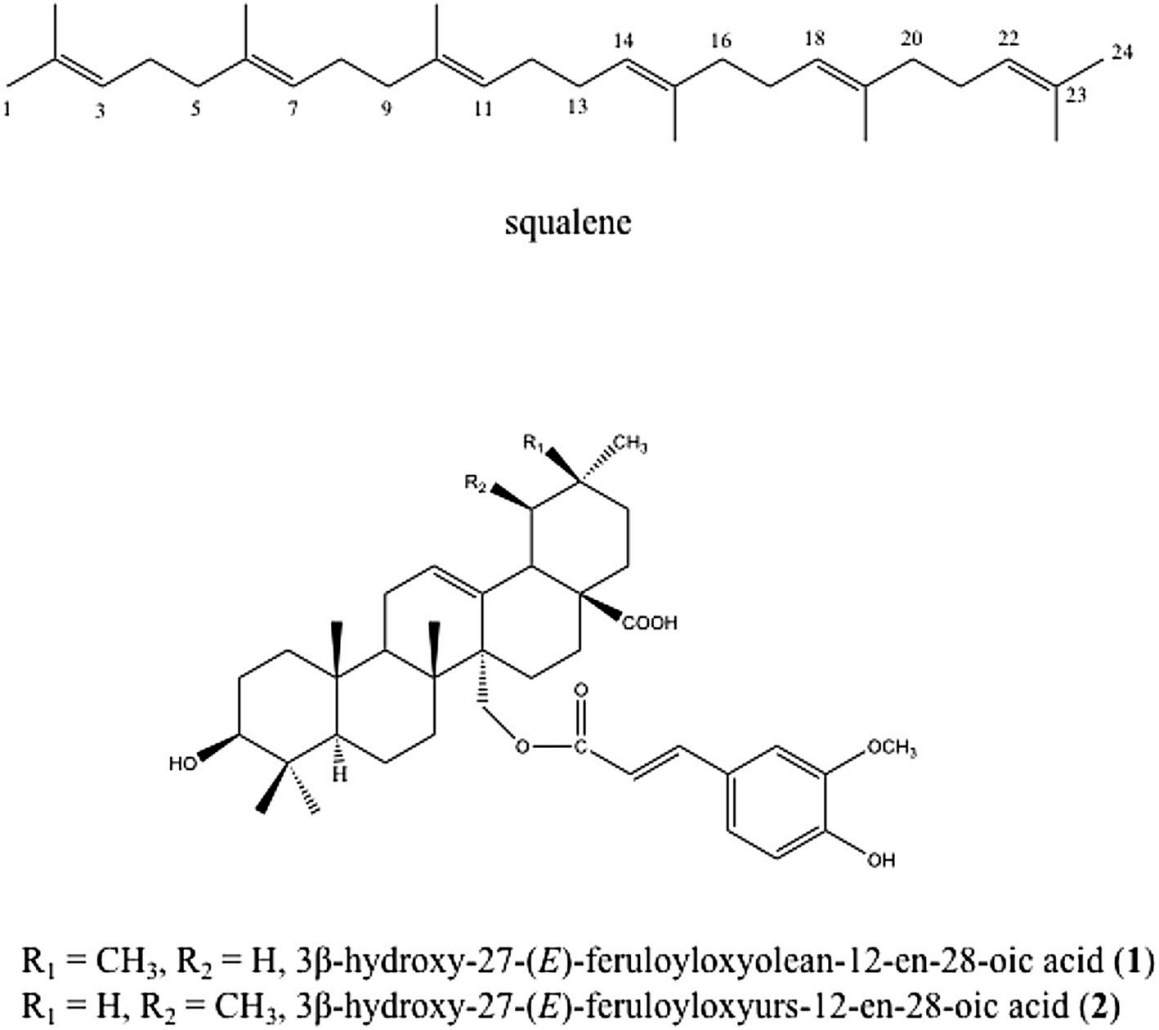
Chemical structures of squalene and two phenylpropanoid conjugated triterpenes, 3β-hydroxy-27-(*E*)-feruloyloxyolean-12-en-28-oic acid (**1**) and 3β-hydroxy-27-(*E*)-feruloyloxyurs-12-en-28-oic acid (**2**)

The correlation between the moieties was detected applying the STOCSY algorithm to the selected signals from the loadings (Fig. 4a). The driver peak at δ 7.56 (*d, J* = 15.9 Hz, 1H, CH) from the H-7 of ferulic acid moiety strongly correlated with many terpenoidal signals, as well as other signals of ferulic acid including OCH_3_. The structural confirmation was based on the NMR data of the eight triterpenic esters with phenylpropanoid moieties isolated from *K. leucantha* twigs by Bero et al. (2013) as cited above. Two-dimensional J-resolved, HSQC and HMBC data for all the above signals indicate the presence of two phenylpropanoid conjugated triterpenes, 3β-hydroxy-27-(*E*)-feruloyloxyolean-12-en-28-oic acid **(1)** and 3β-hydroxy-27-(*E*)-feruloyloxyurs-12-en-28-oic acid **(2)** as the major bioactive compounds present in *Keetia* plants (Fig. 5).

In addition, the signals of squalene in the congested region were also detected and confirmed by STOCSY (Fig. 4c). OPLS–DA revealed that these signals were strongly correlated to *Keetia* samples that displayed low antiplasmodial activity, in which squalene appeared as a major metabolite. The driver peak at δ 1.59 (s, 3H, CH_3_ attached to C-6) showed a high covariance and correlation with the olefinic signals at δ 5.11 (*t, J* = 7.2 Hz, 1H, H-7) and δ 5.15 (*t, J* = 3.8 Hz, 1H, CH, H-11), indicating the presence of three olefinic signals with cis isomerism. Moreover, CH_3_ attached to C-6 also displayed high correlation with the multiplet at δ1.94–2.10 and three singlets at δ1.66, δ1.67, and δ1.60, connecting the olefinic signal with the complex aliphatic structure. Squalene confirmation was performed by 2D-NMR and comparison with data previously reported studies (Rotondo et al. 2017) (Fig. 5).

By comparing the STOCSY obtained through the driver peaks of compounds 1, 2 (Fig. 4a, b), it was possible to observe that both metabolites shared the same pathway, concluding that if the concentration of compound 1 increases, the level of compound 2 will also increase. These results were corroborated by the loadings obtained with the multivariate data analyses based on the IC_50_ values that showed that both these substances contribute synergistically to the increase of the antiplasmodial activity. On the other hand, when the STOCSY plot from the squalene was compared with that of compound 1 and 2 (Fig. 4a–c) it was possible to observe that these compounds were displayed in opposite pathways, representing both a chemical (STOCSY) and a biological (IC_50_) antagonistic effect between squalene and the cyclic triterpenes.

## Conclusion

^1^H NMR metabolic fingerprinting was applied as a high-throughput method for the detection of chemical differences between *Keetia* samples (and their tissues) revealing two major metabolic groups, triterpenoids and phenylpropanoids. Unsupervised PCA analysis of the ^1^H NMR data showed that the effect of tissues was higher than the effect of species on the metabolome and that characteristic triterpenoid methyl signals were more correlated to the twigs of both *Keetia* species, showing higher level of triterpenoids than the leaves. Supervised OPLS–DA based on *Keetia* species (*K. leucantha* and *K. venosa*) showed a very clear separation and a validated model. The resonances of triterpenoids were found to be higher in *K. leucantha* samples than *K. venosa*. In addition, some aromatic resonances were also more correlated with *K. leucantha* and were elucidated as moieties of dihydroxy cinnamic acid derivatives of two triterpenic esters **(1–2)** previously reported in this species by Bero et al. (2013).

To assess the antimalarial potential of the twigs and leaves, all *Keetia* samples were tested for their antiplasmodial activity. In vitro biological results showed that, overall, *K. leucantha* has a similar or a little higher antimalarial activity than *K. venosa* against both chloroquine-sensitive (3D7) and chloroquine-resistant (W2) *P. falciparum* strains. However, in the case of tissues for a same species, there was not much difference and the activity depended on the tested sample. The ^1^H NMR data of *Keetia* samples were correlated with the IC_50_ by OPLS modeling with two Y variables (IC_50_ of 3D7 and W2 strains). To optimize the model, two scaling methods (UV and Pareto) with Log transformation were evaluated and the highest Q2 value was obtained when UV scaling with log transformation of Y variables was used. A strong correlation between metabolomics data and antiplasmodial results was found in the score plot of OPLS modeling. Furthermore, characteristic signals of triterpenoids, such as methyls, H-5 around δ 3.2 and H-12 around δ 5.2 were clearly related with the activity, as well as some ^1^H resonances of phenylpropanoids.

Overall, NMR-based metabolomics combined with supervised multivariate data analysis has proved to be a powerful strategy for the identification of bioactive metabolites in plant extracts. However, the difficulty of hyphenating NMR with chromatographic separation systems and its low-sensitivity result in spectral congestion and hamper the identification of convoluted metabolites, such as triterpenes. In this study, these problems have been solved by successfully combining STOCSY with 2D-NMR, allowing a detailed analysis of different triterpenes in the raw-extract, overcoming structure similarity and coalescence in the aliphatic region.

Although STOCY-supported NMR-based profilins could give a large improvement for the identification of metabolites, there are still limitations to fully identify a metabolite in mixtures due to high congested signals and similarity between analogues of a certain metabolites, e.g. terpenoids or steroids in secondary metabolites. These shortcomings could be overcome by a development of specific preparative methods for the isolation, which might be supported by mass spectrometry confirmation.

## Electronic supplementary material

Below is the link to the electronic supplementary material.

Supplementary material 1 (PNG 142 KB)

Supplementary material 2 (PNG 177 KB)

Supplementary material 3 (PNG 137 KB)

Supplementary material 4 (PNG 133 KB)

Supplementary material 5 (XLSX 204 KB)
