# Supplementary figures and images for: Identification of antiplasmodial triterpenes from Keetia species using NMR-based metabolic profiling
Source: Metabolomics. 2019 Feb 21;15(3):27. doi: 10.1007/s11306-019-1487-4 (PMC6394458; doi:10.1007/s11306-019-1487-4)

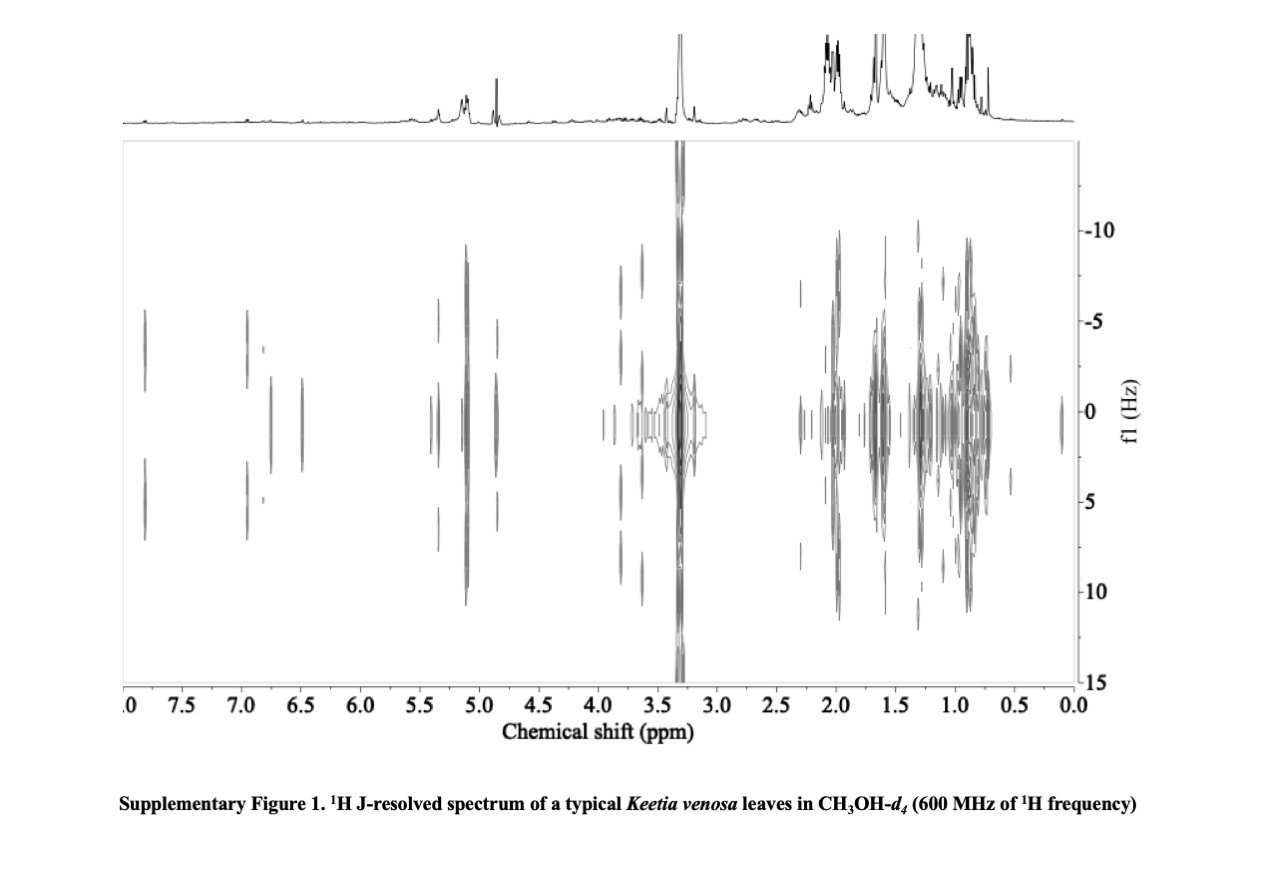

Supplement: Supplementary file 1 — Supplementary material 1 (PNG 142 KB) [file 11306_2019_1487_MOESM1_ESM.png]

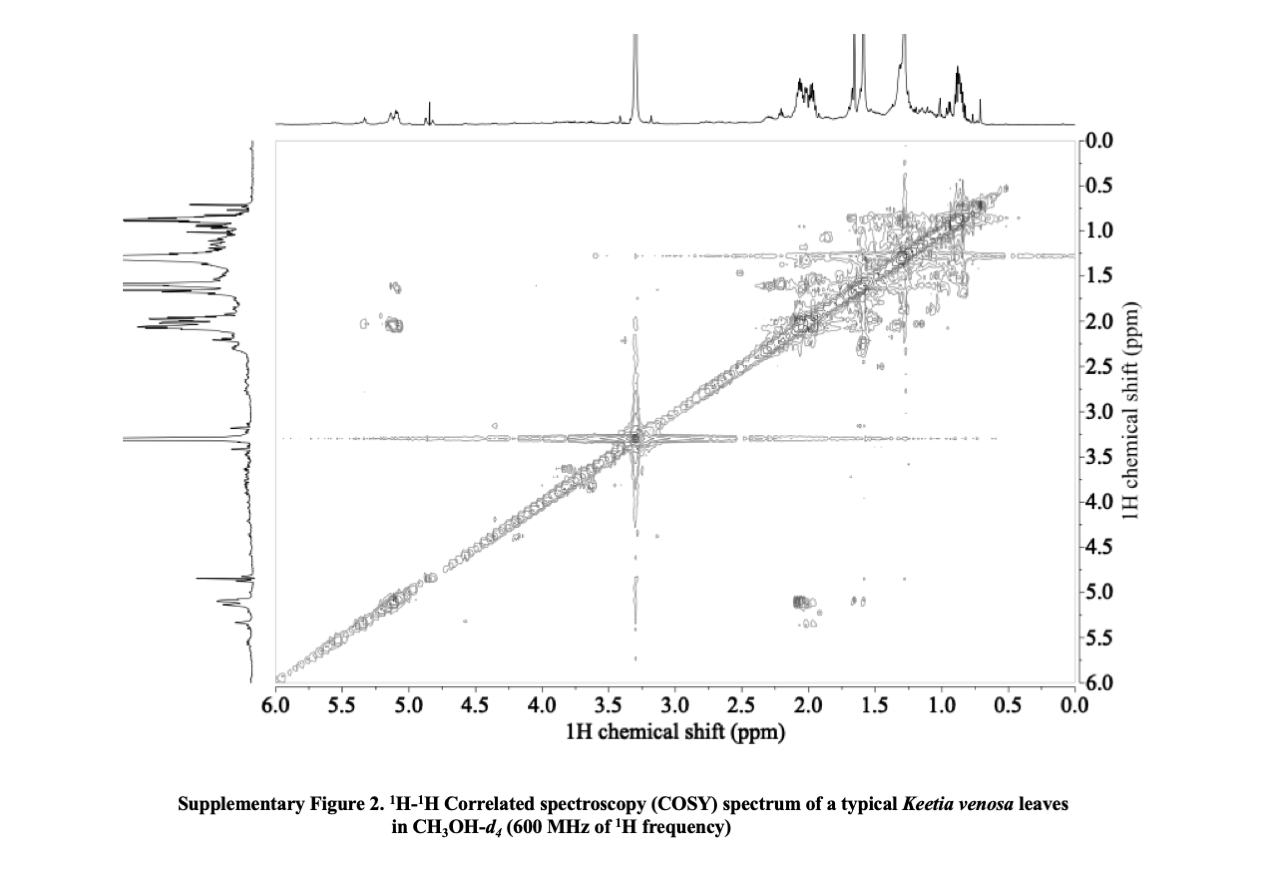

Supplement: Supplementary file 2 — Supplementary material 2 (PNG 177 KB) [file 11306_2019_1487_MOESM2_ESM.png]

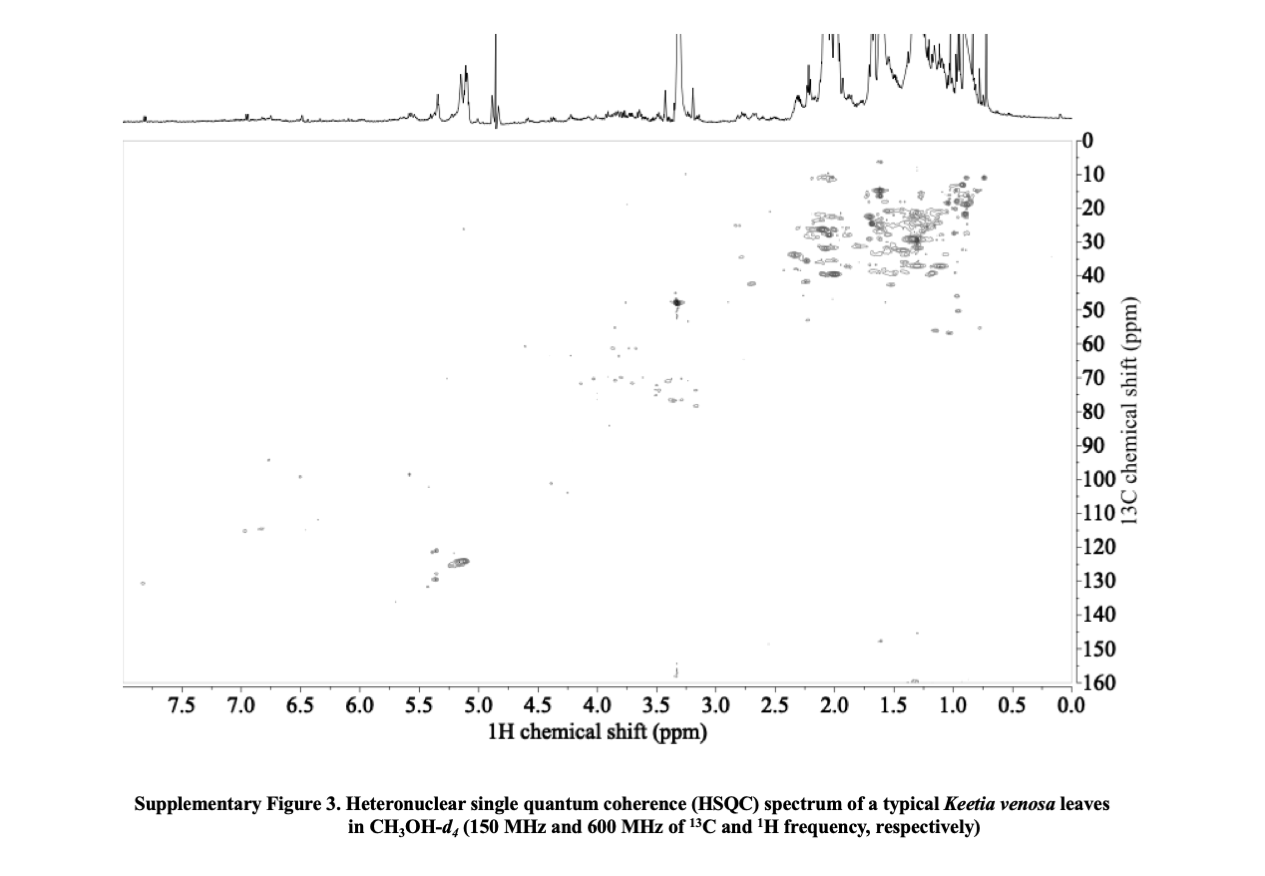

Supplement: Supplementary file 3 — Supplementary material 3 (PNG 137 KB) [file 11306_2019_1487_MOESM3_ESM.png]

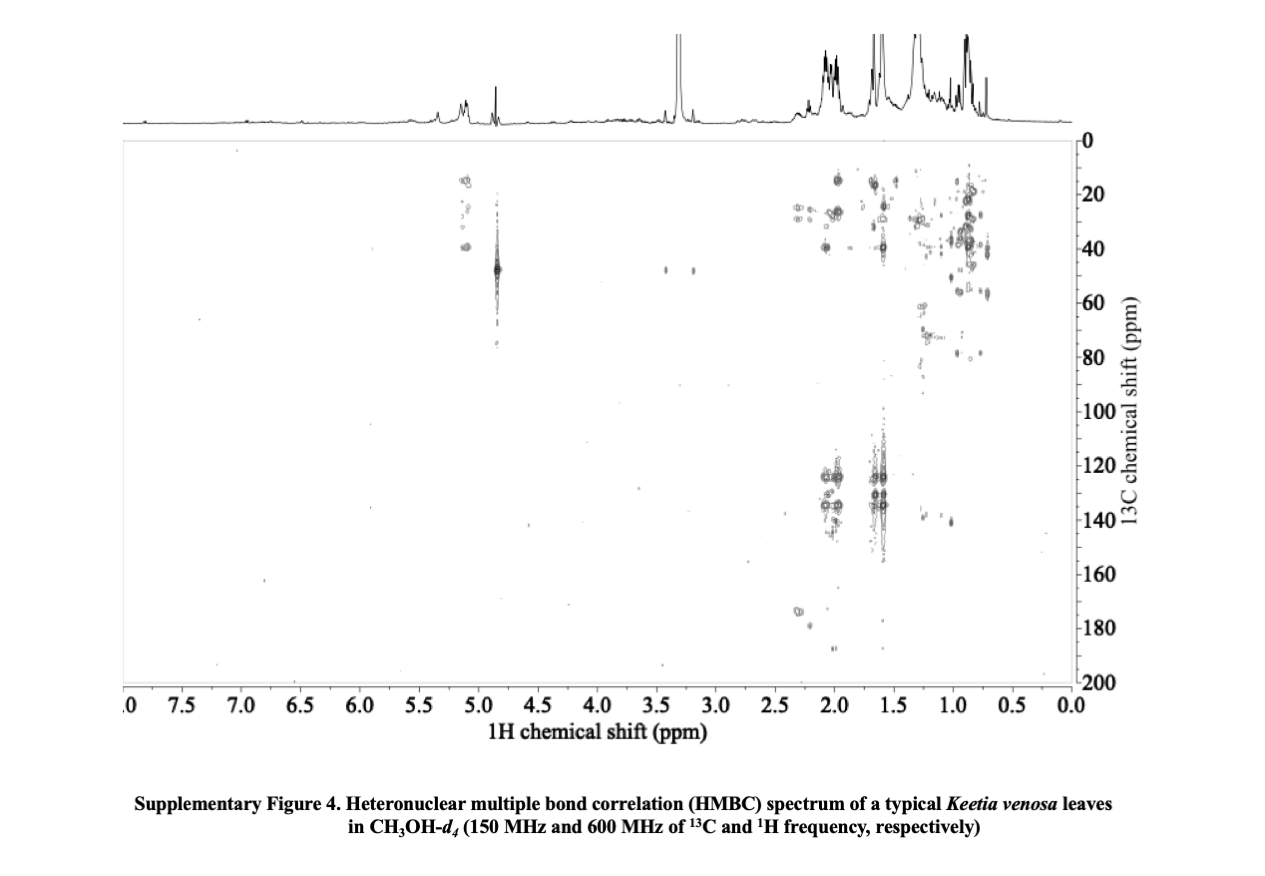

Supplement: Supplementary file 4 — Supplementary material 4 (PNG 133 KB) [file 11306_2019_1487_MOESM4_ESM.png]
